# 

**Published:** 1893-09-09

**Authors:** 


### CONTENTS.

Workers and Weariness - - - - - - - - - 369

Around the Hospitals - - - - - - - - - 370

Great Achievements Under Suffering. III.—The Deaf Musician - - - - - - - - - - 371

Contemporary Theory and Research - - - - - - 372

A Physiological Lantern. II.—The Baby: Its Manners and its Morals - - - - - - - - - - 373

Gleanings in Science and Medicine - - - - - - 374

Annotations.—"My Lord Porridge"—The Laird of Auchentibber—How to Detect Quacks - - - - - - - - 375

Notes and News - - - - - - - - - - 376

Physical Education - - - - - - - - - - 380

The Hospital Clinic—
ROYAL ORTHOPÆDIC HOSPITAL. Treatment of Hammer Toe - 377
THE TREATMENT OF TUBERCULAR ENTERITIS (concluded) - - 378
BRISTOL ROYAL INFIRMARY.—Treatment of Tonsillitis - - 378
NEW DRUGS AND PREPARATIONS. Analgen (concluded) - - 379
The Institutional Workshop—
HOSPITAL ADMINISTRATION.—III.—Special Hospitals: Children's 381
HOSPITAL MEETINGS.—The London Hospital and "The Pall Gazette"—The Middlesex Hospital—The Poplar Hospital for Accidents - - - - - - - - - - - 382
The Book World of Medicine and Science - - - - vii
Medical Vacancies and Appointments - - - - - xi

### EXTRA SUPPLEMENT.—THE NURSING MIRROR.

News from the Nursing World.—Workhouse Sick Wards and Poor Law Infirmaries—Ladies in the Nursery—Wholesome Workers—Fellow Lodgers—Training at St. Marylebone Infirmary—Lord Winchelsea's Scheme—Sickness and Sewerage—Welcome News from Ireland—Gay Doings at Dingwall—A Reasonable Complaint—Australia—A Nursing Home in Madras—True Gentlewomen—Short Items - ccxxxi

The Truth About the London Hospital - - - ccxxxiii

The Work of Deaconesses in Germany - - - - ccxxxiv

Cottage Nurses - - - - - - - - - - ccxxxv

Everybody's Opinion.—Who Supplies the Drugs?—An Answer to an Unprofessional Correspondent — Nurses' Uniform—Tea Drinking - - - - - - - - ccxxxvi

Notes from Philadelpia - - - - - - - - ccxxxvii

News from Australia - - - - - - - - ccxxxvii

For Reading to the Sick.—Prayer—II. - - - - - ccxxxvii

Appointments—Minor Appointments - - - - ccxxxviii

The Muses' Looking Glass.—Womanliness - - - ccxxxviii

Notes and Queries - - - - - - - - - ccxxxviii

The Book World for Women and Nurses - - - - ccxxxix

